# Supplementary material for: Testing for SARS-CoV-2 in resource-limited settings: A cost analysis study of diagnostic tests using different Ag-RDTs and RT-PCR technologies in Mozambique
Source: PLOS Glob Public Health. 2023 Jun 13;3(6):e0001999. doi: 10.1371/journal.pgph.0001999 (PMC10263322; doi:10.1371/journal.pgph.0001999)
Supplement: S1 Table — (DOCX) [file pgph.0001999.s001.docx]

**Identificação de Procedimentos/Actividades durante o Atendimentos de Casos Suspeitos de SARS-CoV-2**

| **Descrição da Actividade** | **Pessoal Involvido (categoria)** | **Qtd.** | **Tempo Gasto p/min** | **Medicamentos e Consumíveis** | **Unidades** | **Observações** |
| --- | --- | --- | --- | --- | --- | --- |
|  |  |  |  |  |  |  |
|  |  |  |  |  |  |  |
|  |  |  |  |  |  |  |
|  |  |  |  |  |  |  |
|  |  |  |  |  |  |  |
|  |  |  |  |  |  |  |
|  |  |  |  |  |  |  |
|  |  |  |  |  |  |  |
|  |  |  |  |  |  |  |
|  |  |  |  |  |  |  |
|  |  |  |  |  |  |  |
|  |  |  |  |  |  |  |
|  |  |  |  |  |  |  |
|  |  |  |  |  |  |  |
|  |  |  |  |  |  |  |
|  |  |  |  |  |  |  |
|  |  |  |  |  |  |  |
|  |  |  |  |  |  |  |
|  |  |  |  |  |  |  |
|  |  |  |  |  |  |  |
|  |  |  |  |  |  |  |
|  |  |  |  |  |  |  |
|  |  |  |  |  |  |  |
|  |  |  |  |  |  |  |
|  |  |  |  |  |  |  |
|  |  |  |  |  |  |  |
