# Supplementary material for: Testing for SARS-CoV-2 in resource-limited settings: A cost analysis study of diagnostic tests using different Ag-RDTs and RT-PCR technologies in Mozambique
Source: PLOS Glob Public Health. 2023 Jun 13;3(6):e0001999. doi: 10.1371/journal.pgph.0001999 (PMC10263322; doi:10.1371/journal.pgph.0001999)
Supplement: S2 Table — (DOCX) [file pgph.0001999.s002.docx]

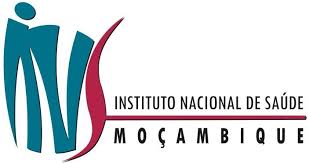


**Avaliação de novas tecnologias e amostras alternativas para o diagnóstico da SARS-CoV-2**

1. **Recursos Humanos**

Para a obtenção da tabela salarial (salário mensal, incluindo benefícios) do pessoal (directa ou indirectamente) envolvido no processo de testagem. ***Esta informação serve para estimar o custo do tempo gasto* (**Tabela 1**)**.

***Tabela 1*: Tabela de Salário Mensal do Pessoal Envolvido**

| **Categoria dos Funcionários** | **Salário Mensal** | **Regalias / Subsídios** | **Total** |
| --- | --- | --- | --- |
| Técnico de Medicina Geral |  |  |  |
| Técnico de Laboratório |  |  |  |
| Técnico de Medicina Preventiva |  |  |  |
| Agente de Serviços |  |  |  |
|  |  |  |  |


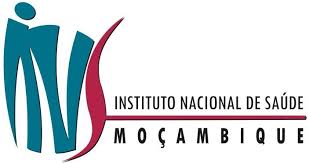


**Avaliação de novas tecnologias e amostras alternativas para o diagnóstico da SARS-CoV-2**

1. **Contabilidade**

Dados sobre **Gastos Gerais**.

| **Tabelas de Custos Anuais - 2020** | |  |
| --- | --- | --- |
|  |  |  |
| **ITEM** | **Sub-item por Custear** | **Custo Anual por Item** |
| **Serviços de**  **terceiros** | Taxas de: |  |
|  | Água |  |
|  | Electricidade |  |
|  | Telefone |  |
| **Veículos** | Manutenção |  |
|  | Combustíveis e lubrificantes |  |
| **Artigos Diversos** | Gastos por: |  |
|  | Material de escritório, impressão e encadernação |  |
|  | Material de Limpeza e Higiene (detergentes) |  |
| **TOTAL** | |  |
|  |  |  |
| Custo da(s) campanha(s) ou reprodução de material publicitário sobre a COVID-19 no ano financeiro 2020 | |  |

| Custo inicial de treinamento de pessoal (para colheita de amostras, gestão de casos suspeitos, gestão de dados |  |
| --- | --- |


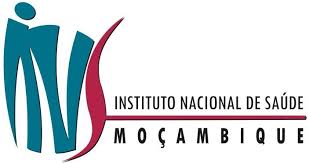


**Avaliação de novas tecnologias e amostras alternativas para o diagnóstico da SARS-CoV-2**

1. **Núcleo de Estatística e Planificação** (NEP)

Desempenho anual – 2020

| **Tabela de Desempenho (produção) Anual** | |
| --- | --- |
|  |  |
| **ITEM** | **Qtd.** |
| Número de utentes (no geral) atendidos na US em 2020 |  |
| Ambulatório |  |
| Internamentos |  |
|  |  |
| Número de utentes testados em 2020 |  |
| Para SARS-CoV-2 |  |
| Para outras patologias |  |
|  |  |
