# Supplementary material for: Testing for SARS-CoV-2 in resource-limited settings: A cost analysis study of diagnostic tests using different Ag-RDTs and RT-PCR technologies in Mozambique
Source: PLOS Glob Public Health. 2023 Jun 13;3(6):e0001999. doi: 10.1371/journal.pgph.0001999 (PMC10263322; doi:10.1371/journal.pgph.0001999)
Supplement: S3 Table — (DOCX) [file pgph.0001999.s003.docx]

**ACTIVOS DE CAPITAL**

| **Mobiliário** | | **Equipamento Médico** | | **Equipamento Não -Médico** | | **Edifícios** | | | |
| --- | --- | --- | --- | --- | --- | --- | --- | --- | --- |
| **Nome do Item** | **Qtd.** | **Nome do Item** | **Qtd.** | **Nome do Item** | **Qtd.** | **Nome do Local** | **C** | **L** | **Área^2^** |
|  |  |  |  |  |  |  |  |  |  |
|  |  |  |  |  |  |  |  |  |  |
|  |  |  |  |  |  |  |  |  |  |
|  |  |  |  |  |  |  |  |  |  |
|  |  |  |  |  |  |  |  |  |  |
|  |  |  |  |  |  |  |  |  |  |
|  |  |  |  |  |  |  |  |  |  |
|  |  |  |  |  |  |  |  |  |  |
|  |  |  |  |  |  |  |  |  |  |
|  |  |  |  |  |  |  |  |  |  |
|  |  |  |  |  |  |  |  |  |  |
|  |  |  |  |  |  |  |  |  |  |
|  |  |  |  |  |  |  |  |  |  |
|  |  |  |  |  |  |  |  |  |  |
|  |  |  |  |  |  |  |  |  |  |
|  |  |  |  |  |  |  |  |  |  |
|  |  |  |  |  |  |  |  |  |  |
|  |  |  |  |  |  |  |  |  |  |
|  |  |  |  |  |  |  |  |  |  |
|  |  |  |  |  |  |  |  |  |  |
|  |  |  |  |  |  |  |  |  |  |
|  |  |  |  |  |  |  |  |  |  |
|  |  |  |  |  |  |  |  |  |  |
|  |  |  |  |  |  |  |  |  |  |
|  |  |  |  |  |  |  |  |  |  |
|  |  |  |  |  |  |  |  |  |  |
|  |  |  |  |  |  |  |  |  |  |
|  |  |  |  |  |  |  |  |  |  |
