# Supplementary material for: Testing for SARS-CoV-2 in resource-limited settings: A cost analysis study of diagnostic tests using different Ag-RDTs and RT-PCR technologies in Mozambique
Source: PLOS Glob Public Health. 2023 Jun 13;3(6):e0001999. doi: 10.1371/journal.pgph.0001999 (PMC10263322; doi:10.1371/journal.pgph.0001999)
Supplement: S4 Table — (DOCX) [file pgph.0001999.s004.docx]

Statistical Annex

**Table A**. Cost components for the estimation of Unit Costs of diagnosing by Nasopharyngeal tests*: Panbio COVID-19 Ag rapid test (Abbott. Jena. Germany. Ref: 41FK10 Lot: 41ADF115A) and STANDARD Q COVID-19 Ag test (SD Biosensor. Suwon-si. South Korea. Ref: Q-NCOV-01G Lot: QCO3020169I)

| **Type of Cost** | **Item** | **CS Marracuene** | **HG Chamanculo** | **HG Mavalane** | **HP Matola** |
| --- | --- | --- | --- | --- | --- |
| *Direct Medical Cost* | Personnel | 1.2 | 1.1 | 1.4 | 3.9 |
|  | Supplies | 6.3 | 7.8 | 6.9 | 8.6 |
| *Direct non-Medical Cost* | Overhead | 0.2 | 3.4 | 1.8 | 2.2 |
|  | Capital | 0.8 | 1.0 | 0.3 | 0.5 |
| ***TOTAL*** | | 8.5 | 13.3 | 10.4 | 15.2 |

* NOTE: The Ag-RDT devices’ cost and the screening standards procedure for both nasopharyngeal tests are the same. This is the reason why. they were put in the same table.

**Table B**. Cost components for the estimation of Unit Cost of diagnosing by Panbio COVID-19 Ag rapid diagnostic test device nasal (Abbott. Jena. Germany. Ref: 41FK11 (***Nasal***)

| **Type of Cost** | **Item** | **CS Marracuene** | **HG Chamanculo** | **HG Mavalane** | **HP Matola** |
| --- | --- | --- | --- | --- | --- |
| *Direct Medical Cost* | Personnel | 1.2 | 1.1 | 1.4 | 3.9 |
|  | Supplies | 3.4 | 4.8 | 3.9 | 5.7 |
| *Direct non-Medical Cost* | Overhead | 0.2 | 3.4 | 1.8 | 2.2 |
|  | Capital | 0.8 | 1.0 | 0.3 | 0.5 |
| ***TOTAL*** | | 5.6 | 10.3 | 7.4 | 12.3 |

**Table C**. Cost components for the estimation of Unit Cost of diagnosing by COVIOS Ag COVID-19 Rapid Antigen Test (Global Access Diagnostics. United Kingdom. Ref: 11811125. Lot: CA25K-121-2)

| **Type of Cost** | **Item** | **CS Marracuene** | **HG Chamanculo** | **HG Mavalane** | **HP Matola** |
| --- | --- | --- | --- | --- | --- |
| *Direct Medical Cost* | Personnel | 1.2 | 1.1 | 1.4 | 3.9 |
|  | Supplies | 7.0 | 8.4 | 7.5 | 9.3 |
| *Direct non-Medical Cost* | Overhead | 0.2 | 3.4 | 1.8 | 2.2 |
|  | Capital | 0.8 | 1.0 | 0.3 | 0.5 |
| ***TOTAL*** | | 9.2 | 13.9 | 11.0 | 15.9 |

**Table D**. Cost components for the estimation of Unit Cost of diagnosing by LumiraDx SARS-CoV-2 Ag Test (LumiraDx. London. UK. Ref.: L016000109048. Lot.: GM2000390)

| **Type of Cost** | **Item** | **CS Marracuene** | **HG Chamanculo** | **HG Mavalane** | **HP Matola** |
| --- | --- | --- | --- | --- | --- |
| *Direct Medical Cost* | Personnel | 1.2 | 1.1 | 1.4 | 3.9 |
|  | Supplies | 7.4 | 8.9 | 8.0 | 9.8 |
| *Direct non-Medical Cost* | Overhead | 0.2 | 3.4 | 1.8 | 2.2 |
|  | Capital | 0.8 | 1.0 | 0.3 | 0.5 |
| ***TOTAL*** | | 9.6 | 14.4 | 11.5 | 16.4 |

**Table** **E**. Cost components for the estimation of Unit Cost of diagnosing by different RT-PCR technologies at INS (COBAS 6800; Abbott m2000sp; QuantStudio 5 or QuantStudio 7 Flex)

| Type of Cost | Item | INS |
| --- | --- | --- |
| *Direct Medical Cost* | Personnel | 2.7 |
|  | Supplies | 34.5 |
| *Direct non-Medical Cost* | Overhead | 1.8 |
|  | Capital | 0.2 |
| *TOTAL* | | 39.2 |
