# Supplementary material for: Testing for SARS-CoV-2 in resource-limited settings: A cost analysis study of diagnostic tests using different Ag-RDTs and RT-PCR technologies in Mozambique
Source: PLOS Glob Public Health. 2023 Jun 13;3(6):e0001999. doi: 10.1371/journal.pgph.0001999 (PMC10263322; doi:10.1371/journal.pgph.0001999)
Supplement: Attachment — Submitted filename: Response to Reviewers_1704.docx [file pgph.0001999.s006.docx]

Nádia Sitoe

Instituto Nacional de Saúde (INS)

Endereço: EN1, Bairro da Vila – Parcela nº3943

Distrito de Marracuene

Província de Maputo - Moçambique

[nadia.sitoe@ins.gov.mz](mailto:nadia.sitoe@ins.gov.mz)

# **Academic Editor’s comments**

1. Please amend your detailed online Financial Disclosure statement. This is published with the article. It must therefore be completed in full sentences and contain the exact wording you wish to be published.

(a) State the initials, alongside each funding source, of each author to receive each grant. For example: "This work was supported by the National Institute of Health (####### to AM; ###### to CJ) and the National Science Foundation (###### to AM)."

Our response: We have now included this statement in page 15.

b) State what role the funders took in the study. If the funders had no role in your study, please state: “The funders had no role in study design, data collection and analysis, decision to publish, or preparation of the manuscript.”

Our response: Please note this statement was already included in the original manuscript in page 15.

2. Please update your online Competing Interests statement. If you have no competing interests to declare, please state: “The authors have declared that no competing interests exist.”

Our response: We have now included the Competing interest’s statement in page 15.

3. Please provide a complete Data Availability Statement in the submission form, ensuring you include all necessary access information or a reason for why you are unable to make your data freely accessible. If your research concerns only data provided within your submission, please write “All data are in the manuscript and/or supporting information files” as your Data Availability Statement.

Our response: The costing dataset has now been uploaded as Supplementary file 4. We have now included such a statement in page 15.

4. Please provide separate figure files in .tif or .eps format only and ensure that all files are under our size limit of 10MB. For more information about how to convert your figure files please see our guidelines: <https://journals.plos.org/globalpublichealth/s/figures>

Our response: As suggested, we now provided Figure 1 and Figure 2 in .tif format in a separate file.

# Reviewer 1' comments:

Reviewer #1: Overall Recommendation
Interesting study calculating the cost of rapid antigen diagnostic testing and RT-PCR for SARS-CoV-2 in Mozambique. The major findings are that the average cost of Ag testing was ~13 USD, compared to ~39 USD for RT-PCR. I think these results are interesting and informative for policymaking, however, as it stands, I do think the analysis needs some fine-tuning, the methods clarified, and the discussion reframed prior to publication.

My major concerns (with detailed explanations below) are as follows: 1) I don’t fully understand why nasopharyngeal and nasal rapid antigen tests are divided. In practice, these procedures (and as evidenced by the costs in this study), are similar.

Our response: We thank the reviewer for this insightful observation. Indeed, the screening standard procedure per healthcare facility, using whatever RDT type, is the same; what differs is the cost of RDT device what make the mean unit costs almost similar. However, we deemed it important to separate since the use of Nasopharyngeal and Nasal in Mozambique was implemented in different periods (June-November 2021 and December 2021 to present, respectively), and we intend to show the cost of each type of RDT used over the COVID-19 management period and later on allow future studies (e.g., cost effectiveness studies) to have the detailed costs of each type of test. We have now added this explanation in the Introduction, page 3.

2) The major driver of cost seemed to be medical supplies, followed by personnel, yet there is very little commentary in the study regarding the operational system in which testing took place, which to me is critical.

Our response: We accept this suggestion. Describing the operational system in which testing took place will make the readers dive into the Mozambican testing reality. We have now included an explanatory sentence in the introduction (pages 3 & 4) describing the scenarios.

3) In the methods, there are several supplies that were included that seem like they should not have been,

Our response: Thanks for spotting this. Since the patients had to test for HIV and malaria before testing to COVID-19, we thought these device costs should be included. We realise these are unnecessary for our objective and have now removed them from the costing exercise (as well as from the Table S2 – Supplementary tables). Please note this has now led to a decrease in the mean costs.

[… and I also don’t see clear documentation as to how capital and overhead costs were calculated.

Our response: As suggested, we have now clarified the costing procedure for both overhead and capital costs (*page 8 in “Direct Non-Medical Costs”*). The details of the capital cost calculations can be found in the Supplementary file 5_Dataset.

In one section, it says that building costs were included, yet in another section it says that “screening for SARS-CoV-2 was performed in tents isolated from the main hospital buildings.”

Our response: We accept our original explanation may have caused confusion. Both buildings and tents were used, however, the latter was predominant. We have now clarified this point in several parties of the paper (such as in page 3 in the introduction; page 8 “under capital costs”; and in the result section page 11 “Overhead & capital costs”.

In the results, this translates to the overhead/capital costs of a national lab being similar to that of a primary health center, which I find hard to believe.

Our response: We understand the reviewer’s concern, but we would also like to bring to your attention a few reasons why buildings and overhead costs contribute less in the unit cost at the INS: it might be related to (a) the fact that most tests in larger hospitals were carried out in separate, low-value tents (see our response above); (b) the value of infrastructures to be rather low in Mozambique, due to the old age of the hospitals under analysis and the high depreciation of capital, and (c) the number of tests performed in the year of analysis (the higher the denominator, the lower the result).

4) Figure 2 in the sensitivity analysis needs re-working, as it’s very challenging to interpret that part of the analysis.

Our response: We have now overhauled the sensitivity analysis section, and the new Figure 2 now presents costs under 3 different scenarios where we varied personnel and medical supplies – see “Figure 2” and descriptions of Sensitivity Analysis section in pages 13, 14, and 15 (Discussion).

5) clarify whether these are costs for diagnosis of asymptomatic/screening purposes, or for symptomatic SARS-CoV-2, as the costs are vastly different.

Our response: These costs are for diagnosis of symptomatic SARS-CoV-2. We have now clarified this point in three passages, namely: page 2 in “Abstract – Background”, and pages 3 and 4 “Introduction”.

6) the discussion needs reframing to highlight the points listed below.

Detailed Recommendations
Abstract
-double or 1.5-space this for easier editing.

Our response: We completely redrafted the Discussion to streamline the key messages, and have now used 1.5 space as recommended (Discussion, pag.12-13).

-“Two years after the onset…” – change this to reflect that we are now 3 years out from the start of the pandemic.

Our response: We have corrected the time as recommended. This was done in the “Abstract – Background”, page 2.

-“the reverse…” “the antigen…” – “the” is not necessary.

Our response: This has now been corrected.

-“2020-21…” – Specify which dates in 2020 – 2021 the study took place, given how dynamic the RDT landscape was throughout this period.

Our response: We thank the reviewer for spotting this. The study took place from November 2020 to December 2021. We have now stated the interval with months in the “Abstract – Introduction”, page 3).

-“four health facilities, primary, secondary and tertiary levels…” – I’m not sure what primary/tertiary levels are. Are you reference to healthcare facility levels? If yes, would clarify.

Our response: We are here referring to the levels of a healthcare systems, that in Mozambique is divided into primary care facilities, secondary rural, and tertiary for high complexity care in urban areas. We have now introduced this definition of the Mozambican healthcare facilities’ levels from the 2014-2019 Health Sector Strategic Plan and added in the “Study Setting” (page 5).

-“a RT-PCR” – the “a” is extraneous here.

Our response: We have now corrected accordingly (“Abstract – Methodology”, page 2).

-“Shown” -> “show”

Our response: We have now corrected accordingly (“Abstract – Principal fundings”, page 2).

-the word “average” should be changed to “mean” throughout the manuscript.

Our response: We have replaced the word as suggested throughout the manuscript.

-was USD 12.90 the mean for both NP Ag-RDTS? Space-permitting, would list the specific cost for each of the two NP Ag-RDTs in the same way that you did for the nasal RDTs.

Our response: Following this recommendation, we have now presented the cost for each NP Ag-RDT in the Abstract – Findings”, page 2.

-“the average unit cost regardless the type…” – there is an “of” missing here.

Our response: This has now been corrected as recommended (Abstract – Fndings”, page 2).

-“Ag-RDT is USD 12.70” – this should be a “was”, not “is”

Our response: This has now been corrected as recommended (“Abstract – Principal fundings”, page 2).

-“costed” -> “cost”

Our response: This has now been corrected as recommended (“Abstract – Principal fundings”, page 2).

-“Ag-RDTs were three times” – this should say “was three times”

Our response: This has now been corrected as recommended (“Abstract – Conclusions”, page 2).

-“analysis” -> “analyses”

Our response: This has now been corrected as recommended (“Abstract – Conclusions”, page 2)

-clinical performance would influence the cost-effectiveness of the test, but not the cost, per se, so I would change this. The setting in which the test takes place could influence the cost.

Our response: This has now been corrected as recommended, namely: The terms *“clinical performance of the Ag-RDTs and epidemiological context***”**, were already removed from the “Abstract – Conclusions”, page 2 and “Discussion” page 14. In the Abstract-Conclusions, the statement remaining is “Additional analyses are recommended as the costs of testing can be influenced by the sample referral system”.

-add the trademark or registered symbol to all names of tests.
Our response: We have now added TM and © as suggested (page 6 “Measurement of Costs”).

Introduction
-“We found….Massachusetts.” – this entire section can be moved to the discussion.

Our response: We agree with your observation, and we have now moved it to “Discussion” (page 12-13).

Materials and Methods
-“Material” -> “Materials”

Our response: We have now corrected this as recommended (page 5).

-please list and describe what RT-PCR test was evaluated, since there is variability in cost between these tests. I now see that this is listed later, but it would be helpful to list this early on, in conjunction with the RDTs.

Our response: We have now reported the RT-PCR technologies following the Ag-RDTs (page 6 “Measurement of Costs”).

-“For the later…” – should be “latter”

Our response: This has been corrected as recommended (page 6 “Measurement of costs”).

-“United State Dollar” – should be “States”

Our response: This has been corrected as recommended (page 6 “Measurement of costs”).

-Tables S1-S4: why are the Panbio and Standard Q tests grouped together in table S1, whereas the other 3 are not? I would presume that the supply cost for these two tests are different, in the same way that it is for the nasal tests? If not, this needs to be clarified.

Our response: This is a useful request for clarification. Both nasopharyngeal (Panbio and Standard Q devices) have the same market price and standard operation procedure (SOP) in each healthcare facility is the same, and they ended up having the same mean unit cost for testing. This is why we opted to put them in the same table to avoid repetition. The specific Tables for specific tests have now been moved to the Supplementary file_4 Statistical Annex, (Tables 1-5).

-Where is the table similar to S1-S4 with information on the RT-PCR?

Our response: We apologise for this lapse. The specific Tables for specific tests have now been moved to the Supplementary file_4 Statistical Annex (Tables 1-5).

-For these S1-S4 tables, to me these seem to be results, not methods, so consider putting this in the results section.

Our response: We agree with this suggestion. These tables present the results of resource consumption per Ag-RDT type per healthcare facility. It makes sense to put them under the result section. We have now the Tables for specific tests have now been moved to the Supplementary file_4 Statistical Annex, (Tables 1-5).

-Supplies and RDTs – I’m not sure why the section regarding HIV and malaria testing is relevant. Consider removing. Similarly, is there a reason why malaria and HIV RDT kits are included in table S6 as a supply cost? I would remove these since you’re just evaluating the cost of covid rdts. This would imply an evaluation of a program to diagnose febrile illness with RDTs, which is not the point of this study.

Our response: We have now removed these costs following the recommendation in number “3” above. As stated in number 3 “… we have removed them from the costing exercise … what led to decrease in the mean costs”.

-Capital Costs: somewhere, please specify whether this is diagnosis of symptomatic SARS-CoV-2, or asymptomatic screening since these protocols are different. For example, I note that in table S6, a thermometer, sphygmomanometer, stethoscope, oximeter, and weighting scale are listed as equipment. This equipment would be relevant for symptomatic patients who would require vital signs, but wouldn’t be necessary in the setting of asymptomatic community screening for example. Similarly, a lot of the capital costs may not be necessary in the setting of mobile community testing for example, where you wouldn’t need a building. This should be noted early as since it is a crucial point.

Our response: We accept this request to clarify whether this costing exercise is for symptomatic or asymptomatic patients. As explained in number “5“ above, this is diagnosis of symptomatic SARS-CoV-2. We have clarified this in three passages, namely: page 2 in “Abstract – Background” and pages 3 and 4 “Introduction”

-Sensitivity analysis: “access” -> “assess”

Our response: This has now been corrected as recommended (page 9 “Sensitivity analysis”).

Results
-“These findings suggest that Ag-RDT…” – this is a discussion point, not a result, and should be moved to the discussion.

Our response: Following this suggestion we have now transferred the sentence to the discussion section (page 12 “Discussion” – first paragraph).

-Table S5: I’m not sure what the last columns are referring to, the labor cost in mzn and usd. Is this the mean labour cost per minute, averaging that for all the workers? If yes, this needs to be clarified, because there are some individuals, like GTP’s , that are skewing this.

Our response: The last columns refer to the mean labour cost per healthcare facility. It derives from the summation of all “labour cost per cadre”. We first estimated the cost of time of all activities performed by a given worker (Labour Cost per Cadre). Since there are activities which can be performed by different cadres (e.g., cadre A & C), we added the costs of both cadres and divided by 2 to get the mean cost for such activity (*this combination can be seen in the costing exercise sheets “Excel” of each HCF*). Finally, the “Mean Labour Cost per HCF” was obtained through the sum of all “labour cost per cadre” (which includes the mean cost of those activities that can be performed by different cadres, plus the cost of those activities that are performed by a single cadre). The specific Tables for specific tests have now been moved to the Supplementary file_4 Statistical Annex, (Tables 1-5).

-Figure 1: The legend needs to be fixed – “Capital” is listed twice (blue and yellow), so I’m not sure how to interpret this graph.

Our response: The legend for Figure 1 has now been fixed.

-consider consolidating these results overall, specifically the way they are presented. As it stands, there are a lot of subsections with one sentence each, and it’s hard to follow.

Our response: We have now merged some of the sections and subsections following this recommendation, streamlined the tables, and moved some of the Tables for specific tests have now been moved to the Supplementary file_4 Statistical Annex, (Tables 1-5). (page 10 “Results”, page 13 “Personnel and supplies Costs; Overhead and Capital Costs).

“A sensitivity analysis modifying the two main cost drivers led to ranges from SUD 1.2 to USD 3.9….” – please clarify what this is referring to. What test? What facility? Etc.

Our response: We have now overhauled the sensitivity analysis section, and the new Figure 2 now presents costs under 3 different scenarios where we varied personnel and medical supplies – see the section 2.2 Sensitivity Analysis in pages 11 in the Results.

Figure two: I’m not sure what this figure means What is minimum, average, and maximum? This all needs to be clarified.

Our response: Figure 2 has been substantially changed to plot 3 different cost scenarios and capturing variations of personnel and medical supply costs – see the new Figure 2, section 2.2 Sensitivity Analysis in pages 11 in the Results.

Discussion
-as above, epidemiological context and clinical performance do not influence cost. They influence cost-effectiveness, which is beyond the scope of this study.

Our response: We appreciate this comment. As mentioned above we removed the terms “**clinical performance of the Ag-RDTs** and **epidemiological context”,** from the “Abstract – Conclusions”, page 2 and “Discussion” page 14. We only state that “Additional analyses are recommended as the costs of testing can be influenced by the sample referral system”.

-overall, this discussion needs to be reframed once the issues above are addressed. I think that comparing costs in Mozambique to costs from study in nursing homes in Germany is not very informative. Once the above changes are made, more interesting would be to focus this discussion on 1) what are the different costs between the different antigen tests, and between antigen testing and PCR. 2) why are costs higher in certain health settings compared to others (can link this to what the major drivers of cost are), etc. 3) this discussion would give policy makers information regarding how to choose a test, estimate costs, and modify them if needed by adjusting the testing algorithms that are deployed.

Our response: We have now overhauled the Discussion to reflect the changes introduced in our analysis, as well as streamline the key messages and policy options. In particular, we have now focused on the difference in costs between the Antigen and PCR options, on the cost drivers, and on the implications for testing algorithms (Discussion, paragraphs 2, 3, and last but one in pag.13).

# Reviewer 2’s comments

This is a good paper on a topical issue that may guide the choice of diagnostic tests considering the cost of various diagnostic test kits available but may benefit from further review putting into consideration the below comments.
1. The tittle does not identify the study as a cost analysis study. Kindly identify the study in the tittle

Our response: We thank the reviewer for the useful comments provided. We accept the suggestion and changed the tittle to**: Testing for SARS-CoV-2 in resource-limited settings: A cost-analysis study of SARS-CoV-2 diagnostic test using different Ag-RDTs and RT-PCR technologies in Mozambique.**

Specific comments
ABSTRACT
Under the results section, the researcher can consider presenting the costs in local currency and the dollar to attain the aim of estimating the cost for the country’s specific context at the same time allow for comparability using the dollar.

Our response: We agree with the recommendations and have changed the currency accordingly in Abstract and Findings (page 2 “Abstract – Findings; and page 10 “Results”).

INTRODUCTION
Replace the word public contamination with spread – To narrow down the purpose of the introduction of rapid diagnosis that aims to aid in implementing the test-trace-isolate strategy which is essential for the treatment of COVID-19 cases. Reducing public contamination involved a lot of measures such as isolation which is another step after diagnosis.

Our response: The term has now been changed (page 3 “Introduction”).

The research could provide the difference between the Antigen and RT-PCR SARS-CoV-2 by even stating the advantages and disadvantages of using each. To justify the country’s implementation of Ag-RDTs for routine diagnosis of all symptomatic suspected cases as a first-line strategy before the PCR test.

Our response: We have now introduced a longer explanation in the “Introduction” (page 3, *second period – highlighted in blue*). See the text below:

“…RT-PCR requires a well-equipped laboratory with advanced technology and highly trained laboratory technicians, which in low- and middle-income countries (LMICs), including Mozambique, is typically available only in a few national reference laboratories. In addition, in cases of high demand, RT-PCR has limited testing capacity which leads to long turnaround times, rendering it ineffective, since it delays isolation and treatment to contain the spread of the virus. Conversely, Ag-RDTs do not require additional infrastructure or equipment and can be operated at the point of care, with results available in less than thirty minutes…”.

Methodology
Page1: Kindly add more clarifications and the reasons why you have chosen 4 public urban facilities and ignore rural and private facilities. This may compromise the generalizability of these results

Our response. We have now clarified the reasons why we have chosen 4 public urban facilities. Hope this can make the results generalizable (page 5 “Materials and Methods – Study Settings”).

Page4: The list and details of the 5 Ag-RDTs should appear in the methodology not in the background. Remove it from introduction to avoid unnecessary repetition.

Our response: We have now removed the details on the Ag-RDTs from the introduction, added information on the RT-PCR technologies (as the reviewer 1’s recommendation), and put both in page 6 (“Measurements of Costs”).

Page5: There is a confusion here between direct non-medical costs and indirect costs. Indirect costs refer to productivity loss and this can be only assessed using a societal perspective yet this study uses payer perspective. Kindly clarify this.

Our response: Thank you for spotting this. We have now deleted the sentence “this is often referred as indirect costs in the literature”. However, we highlighted the already existing sentence which states that “Medium- and long-term indirect costs were not considered in this study” (page 7 “Measurements of Costs”).

It would be great to include a ‘unit’ cost typology to summarize the resource use measurement of the study. For example, including intervention ‘unit’ costs, direct and ancillary service ‘unit’ costs, activity costs, and input costs in the topology.

Our response: Perhaps we had not explained comprehensively what we meant for ‘unit’ in our original submission. In this analysis, resource consumption was summarised as personnel, supplies, overhead, and capital costs to easy the comparison between the 4 healthcare facilities (*table 3-7*). However, they include all the unit cost typology presented in the comment above. Personnel cost included the cost of time of all “activities” performed to deliver the intervention. Supply cost included all “input items” used for patient screening. Overhead costs included all “ancillary services” like, electricity, water, and other administrative costs. We have now tried to clarify these concepts through the new text.

For overhead costs, where the researcher divides the total cost of each component by the total annual number of visits (both outpatients and inpatients). The estimated cost can be difficult to generalize since for a bigger facility the formulae for calculating the overhead cost might overestimate the cost of diagnostic compared to a smaller facility with a relatively less operating cost.

Our response: We understand this suggestion; however, please note we took into consideration two factors: **(i)** the facilities under analysis were references for dealing with SARS-CoV-2, and **(ii)** from April 2020 to December 2021, the Ministry of Health of Mozambique had been encouraging people to “stay at home” and visit the nearby healthcare facility (HCF) if really needed. These factors made us deem it reasonable to allocate the overhead based on number of patients attended to, since during this period there was considerable number of patients presenting to this HCF with flu or SARS-CoV-2 related symptoms or declaring having contact with SARS-CoV-2 positive cases.

The methodology is not clear. Kindly clarify the following;
a. What data collection tools used in this study: Please clarify this by setting a paragraph for the same and make it available as a supplementary document.

Our response: We have now brought in a section (pages 6, Section 1.2 Study Design”) where we detail what data collection we used and similarly we provide them as supplementary documents.

b. Expert opinion with key informants? questionnaires, registers consulted? Please clarify these by categorizing them

Our response: Thank you so much. With “Expert opinion with key informants” we meant an interview with the heads of the triage sector/physicians/nurses, and/or lab technicians involved in the screening. They described what procedure were performed to screening patients and what time (in minutes) was needed (page 7 “Direct Medical Costs – Personnel Costs”. The questionnaires were in form of tables with blank spaces to be filled in. We have now clarified this is in pages 5 and 6 “Study Design”.

c. It is not clear how you allocated of labor time? Kindly clarify

Our response: Labour time was obtained through interview to key personnel (providers who delivered diagnosing services for SARS-CoV-2 at the healthcare facilities). All the providers were asked about the time they spent to conclude each activity in the diagnosing process. Minimum and maxim time was requested and from this the average time was estimated. This section can be found in page 7 “Direct Medical Costs – Personnel Costs”.

d. Kindly clarify how you adjusted for inflation (i. e; consumer price index (CPI)), show the formula and explain the source of CPI and which year if applicable

Our response: To adjust for inflation we have used the mean CPI offered by the Bank of Mozambique (BdM) as referred in page 8 “Capital Costs”.

e. Explain how did you do annuitization (Discount rate if applicable?) of assets if applicable

Our response: We calculated the annuitized cost of each capital item by (i) combining its useful life with a discount rate of 10% (offered by the Bank of Mozambique), then (ii) multiplying the result (which is a decimal number) by the replacement cost (market price) of such ite; and then (iii) sum the health facility's total annuitized costs and divided by the respective number of tests performed. For the replacement cost of buildings, we multiplied the cost per square meter (m^2^) by the total area occupied. This procedure is now explained in page 8 “Capital Costs”.

f. How did you count personnel resource use; it is not clear how you did it

Our response: Data on personnel resource use was obtain from the key informants, as following: key informants were asked to detail the resources and the respective amounts/quantities required to screen a patient for each type of test (e.g. personal protective equipment (PPE), laboratory kits and reagents, and other supplies), as well as the number of days/times/patients for which each item could be used (S2-4 Tables, detail the resources used). This section can be found now in page 8 “Supplies and RDTs”

g. The results are presented in a very summarized manner, kindly present a table showing the identification, measurement and valuation of resources in the results as well as data methods and data sources table

Our response: We have now introduced “Table 2” as a summarized table requested by reviewer 2.

Changes introduced: Table 2 is replica of Table 1. We removed the names of the facilities where data was collected and kept the remaining information in different position (page 10 “Results”). If it does not reflect what the reviewer intended, we are willing to change.

h. The researcher could consider stating the major driver for the cost difference among the different AG-RDTs types to avoid speculation in the section.

Our response: We have now added a sentence to complement the pre-existing to show more the cost difference among the different Ag-RDTs. It is stated as following: “In both settings, health facility and laboratory, supplies were the main cost drivers followed by personnel and overhead. Table 2 provides more details on the major driver for the cost difference among the different Ag-RDTs types” (page 10 “Results”).

i. Kindly explain in a separate paragraph how you did the cost analysis.

Our response: We have now developed a subsection in page 9 (Cost Analysis), to explain how the cost analysis was performed.

j. Clarify any assumptions before analysis.

Our response: The steps taken (assumptions) to perform the cost analysis have now been clarified in the subsection “Cost Analysis” (page 9).

DISCUSSION
I wonder what the positivity rate at the time of the study was. Just like the researcher indicates that many factors can influence the cost of SARS-CoV-2 diagnosis such as the epidemiological context. How can the study be compared to other studies (if any exist) based on the epidemiological context in the study period?

Our response: This point was also picked up by reviewer 1. We have now removed this from the “Abstract – Conclusions” since we realised that it bears no relation with the analysis performed.
